# Supplementary material for: Non-Invasive Detection of Biomolecular Abundance from Fermentative Microorganisms via Raman Spectra Combined with Target Extraction and Multimodel Fitting
Source: Molecules. 2023 Dec 27;29(1):157. doi: 10.3390/molecules29010157 (PMC10780171; doi:10.3390/molecules29010157)
Supplement: Supplementary file 1 [file molecules-29-00157-s001.zip › molecules-2743082-supplementary.pdf]

Supplementary Materials

Xinli Li [a], Suyi Li [a *], Qingyi Wu [b, c]

[a] College of Instrumentation and Electrical Engineering, Jilin University, Changchun 130061, Jilin, P. R China.

[b] Changchun Institute of Optics, Fine Mechanics and Physics, Changchun, 130033, Jilin, P. R China.

[c] University of Chinese Academy of Sciences, Beijing, 100049, Jilin, P. R China.

*Correspondence: lsy@jlu.edu.cn; 86-0431-88502363

Table of Contents

1. Bacterium culture and Raman detection …………………………………………………...P2
2. Figure S1 Bacterial synchronization culture experiment and Raman spectrum acquisition …P2
3. Optical system information…………………………………………………………………...P2-3
4. Figure S2 The optical system information of the Raman spectrometer is self-built………....P3
5. Assignments of Raman bands ………………………………………………………………...P3-4
6. Table S1 Raman band assignments of bacterial cells…………………………………........P3-4
7. References……………………………………………………………………………………..P4

1. Bacterium culture and Raman detection

A detailed description of bacterial culture and Raman detection is shown in Figure A1, with key steps below.

Step1. Bacterial cell culture. Take 100 mL of Luria-Bertani medium and 400 μL of E. coli (or yeast) solution and incubate at 25°C on a shaker with relatively sufficient nutrients for 24 hours.

Step2. Growth curve detection. From inoculation, 3 mL of bacterial solution was aspirated at 0, 1, 2, 3, 4, 5, 6, 7, 8, 16, and 24 h of incubation, and the optical density at wavelength 600 nm (OD600) was recorded using a UV spectrophotometer.

Step3. Sample Handling. Aspirate 1 ml of culture solution in a 1.5 ml centrifuge tube, centrifuge at 9000 rpm for 3 min to collect the bacterial sediment, and wash the bacterial sediment with sterile water at 9000 rpm for 3 min, three times. After washing, place the sediment in 1 ml of sterile water and shake well to mix it for use.

Step4. Raman detection. Using a pipette, 1 μL of each mid-layer solution of different culture times was pipetted onto an aluminized glass substrate. After evaporating the liquid, the samples were observed and measured using the confocal Raman spectrometer (Hooke P300, Hooke Instruments Ltd., China) with a 532 nm wavelength excitation laser.

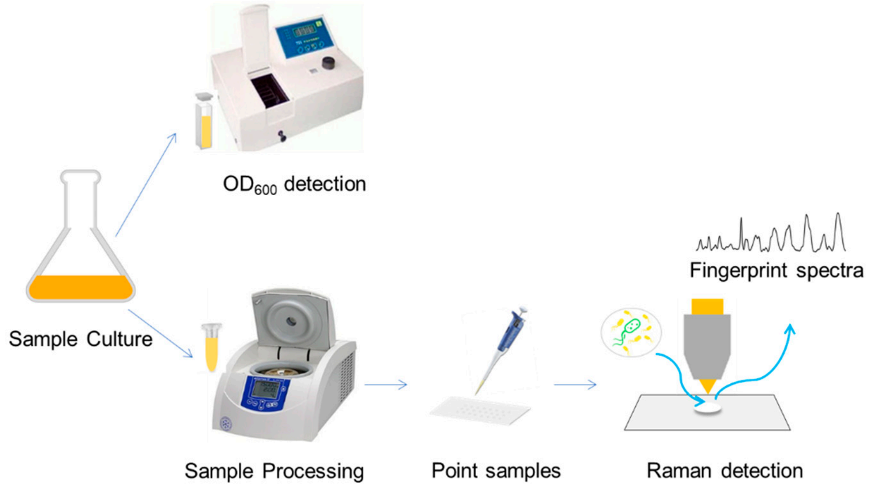

Figure S1. Bacterial synchronization culture experiment and Raman spectrum acquisition

2. Optical system information

In this work, a confocal Raman spectroscopy system shown in Figure A2 was used for the detection of bacterial samples. A 532 nm laser (08-DPL, 200 mW, Cobolt, USA) was used for Raman excitation, and a dry objective (100 × 0.8 NA, LMPlanFL N, Olympus, Japan) was used for our experiments. The substrate chip for the samples was an aluminum-plated overlay on silica glass, which does not damage the cells to reduce the Raman background signal. The spectrometer (ISOPLANE 320, Princeton Instruments, USA) equipped with a diffraction grating with groove densities of 1200 g/mm and a CCD camera (PIXIS 100B, Princeton Instruments, USA) that can be cooled to -75 °C.

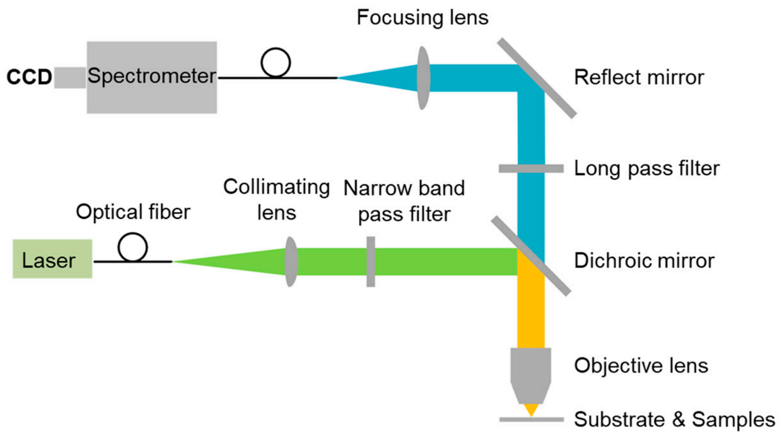

Figure S2. The optical system information of the Raman spectrometer is self-built.

3. Assignments of Raman bands

Table S1 shows the characteristic peak positions extracted in this work, the spectral bands in which they are located and the corresponding intracellular biomolecular vibrations or assignments.

Table S1. Raman bands assignments of bacterial cells

Characteristic peak position (cm-1)	Characteristic peak bonds (cm-1)	Biomolecular vibration	Biomolecular assignment	References
624	[616-626]	C-C twisted of phenylalanine	Protein	[1]
726	[712-732]	A (ring breathing mode of DNA/RNA bases)	Nucleic acid	[2]
760	[758-764]	Tryptophan, δ(ring)	Protein	[3]
786	[772-790]	DNA: O-P-O, cytosine, uracil, thymine	Nucleic acid	[2]
830	[828-836]	Tyrosine (Fermi resonance of ring fundamental and overtone)	Protein	[2]
980	[978-986]	C-C stretching b-sheet	Protein	[1]
1002	[996-1010]	ν(C=C)ar of phenylalanine	Protein	[2]
1034	[1028-1036]	δ(CH) bend of tryptophan and phenylalanine	Protein	[1]
1066	[1062-1068]	PO2- stretching	Nucleic acid	[1]
1096	[1086-1108]	Symmetric phosphate stretch; ν(C-N)	Nucleic acid	[1]

1576	[1562-1582]	Ring breathing modes in the DNA bases G, A	Nucleic acid	[1]
1586	[1586-1598]	Phenylalanine, hydroxyproline	Protein	[1]
1610	[1606-1618]	ν(C=C)ar of tyrosine; ν(C=C) of tryptophan	Protein	[1]

ν: stretching vibration; δ: deformation vibration; ar: aromatic; [L-R]: left and right boundaries of the characteristic peaks.

References

1. A. C. S. Talari, Z. Movasaghi, S. Rehman, I. U. Rehman, Raman spectroscopy of biological tissues, Appl Spectrosc Rev 50(2015) 46-111.
2. R. Mukherjee, T. Verma, D. Nandi, S. Umapathy, Understanding the effects of culture conditions in bacterial growth: A biochemical perspective using Raman microscopy, J Biophotonics 13(2020), e201900233.
3. S. Kloß, P. Rösch, W. Pfister, M. Kiehntopf, J. Popp, Toward culture-free Raman spectroscopic identification of pathogens in ascitic fluid. Anal. Chem. 87(2015) 937-943.
